# Detection and genetic characterization of Uukuvirus lihanense (Uukuvirus, Phenuiviridae) in hard ticks from the Colombian Caribbean

**DOI:** 10.1099/acmi.0.000941.v3

**Published:** 2025-10-13

**Authors:** Ketty Galeano, Yesica López, Camilo Guzmán, Yeimi López, Héctor Contreras, Alejandra Garcia, Luis Romero, Caty Martínez, Daniel Echeverri, Luis Paternina, Alfonso Calderón, German Arrieta, Salim Mattar

**Affiliations:** 1 Instituto de Investigaciones Biológicas del Trópico, Universidad de Córdoba, Córdoba, Colombia1; 2 Universidad de Sucre, Sucre, Colombia

**Keywords:** *Uukuvirus lihanense*, metatranscriptome, hard ticks

## Abstract

Ticks are arthropod vectors that transmit pathogens important to human and animal health. The objective of this work was to identify *Uukuvirus lihanense* in the metatranscriptome of hard ticks. Between October 2022 and June 2023, ticks were collected from rural areas of the Colombian Caribbean area of the departments of Córdoba and Cesar. High-throughput sequencing (next-generation sequencing) was performed using MGI’s DNBSEQ-G50RS. Bioinformatics analyses were performed in Galaxy, diamond and IQ-TREE2. A total of 766 ticks were collected; 87.33% (669/766) were *Rhipicephalus microplus*, 5.4% (42/766) *Dermacentor nitens*, 4.2% (32/766) *Rhipicephalus sanguineus* and 3.0% (23/766) *Amblyomma dissimile*. Complete and partial L and S segments of *Uukuvirus lihanense* (LITV) were detected in the metatranscriptome of *A. dissimile*, *D. nitens* and *R. microplus*. The LITV sequences found are phylogenetically related to those detected in *R. sanguineus* and A. variegatum from the French Antilles, in *R. microplus* from Trinidad and Tobago and *R. microplus* from Brazil. LITV was identified in *D. nitens* and *R. microplus*; the first report was in *A. dissimile*. Although LITV is not considered necessary in public health, the virus belongs to the *Phenuiviridae* family, which includes viruses of public health importance, such as *Dabie banda-virus* and *Bandavirus heartlandense*.

## Data Summary

The authors confirm all supporting data, code and protocols have been provided within the article. Sequences are available in the Sequence Read Archive with accession nos. SRX25381865, SRX25381866, SRX25381867, SRX25381868 and SRX25381869.

## Author Statement

The brief report submitted entitled, ‘Detection and genetic characterization of *Uukuvirus lihanense* (*Uukuvirus, Phenuiviridae*) in hard ticks from the Colombian Caribbean’ and the previously published brief report ‘Hard ticks (Ixodida: Ixodidae) in the Colombian Caribbean harbour the Jingmen tick virus: an emerging arbovirus of public health concern’. They are part of the same research project titled ‘Fortalecimiento de las capacidades de investigación con relación a las enfermedades transmitidas por vectores de las universidades de Córdoba y Cesar 2020–2023 en Córdoba, Cesar’.

## Introduction

Ticks are ectoparasites widely distributed globally and are known as vectors of many viruses important to human and animal health [1,3]. These arthropods host a variety of negative-sense RNA viruses, many of them emerging pathogens, such as the severe fever with thrombocytopenia syndrome virus (*Bandavirus dabieense*) [4] and re-emerging ones such as Crimean-Congo haemorrhagic fever virus, which have caused outbreaks and fatal cases in humans [5].

The genus *Uukuvirus* belongs to the family *Phenuiviridae* [6,10], where critical human pathogens are found, such as *B. dabieense* and *Bandavirus heartlandense* discovered in China and the USA, respectively [11,12]. The genome of Uukuviruses is a negative-sense segmented RNA, with three segments (L, M, S) encoding four proteins. The RNA-dependent RNA polymerase (L), external glycoproteins (Gn and Gc), a nucleocapsid protein (S) and a nonstructural protein (NSs) [6,10]. However, *Uukuvirus lihanense* (LITV) lacks the M segment, a critical component that allows cell entry. This absence of the M segment in LITV is significant as it affects the virus’s ability to infect cells, potentially influencing its pathogenicity and transmission dynamics [5].

Given the vast array of viruses found in ticks, the advent of next-generation sequencing (NGS) technology has revolutionized our ability to investigate and identify tick viromes [3,13,15]. In Colombia, NGS-based studies have recently unveiled the diversity of viruses in ticks, including the genera *Orthoflavivirus*, *Orthonairovirus*, *Bandavirus* and *Uukuvirus*, among others [5,16,19]. The departments of Córdoba and Cesar in the Colombian Caribbean area are particularly significant due to their geographical conditions, diverse reservoirs (such as rodents and birds), vectors (such as ticks and mosquitoes) and predominantly tropical climatic characteristics (high temperatures and humidity), which facilitate the spread of vectors like ticks and the diseases they transmit [20].

Given the increasing threat of tick-borne viruses to public health, it is imperative to conduct eco-epidemiological surveillance using NGS technologies [21].

Our study, which aimed to identify LITV in the metatranscriptome of hard ticks in the Colombian Caribbean, is a timely and important step in addressing the growing threat of tick-borne diseases. The results of the collected tick species were presented in a recently published article [19].

## Methods

### Tick capture, taxonomic identification and RNA extraction

**Fig. 1.**
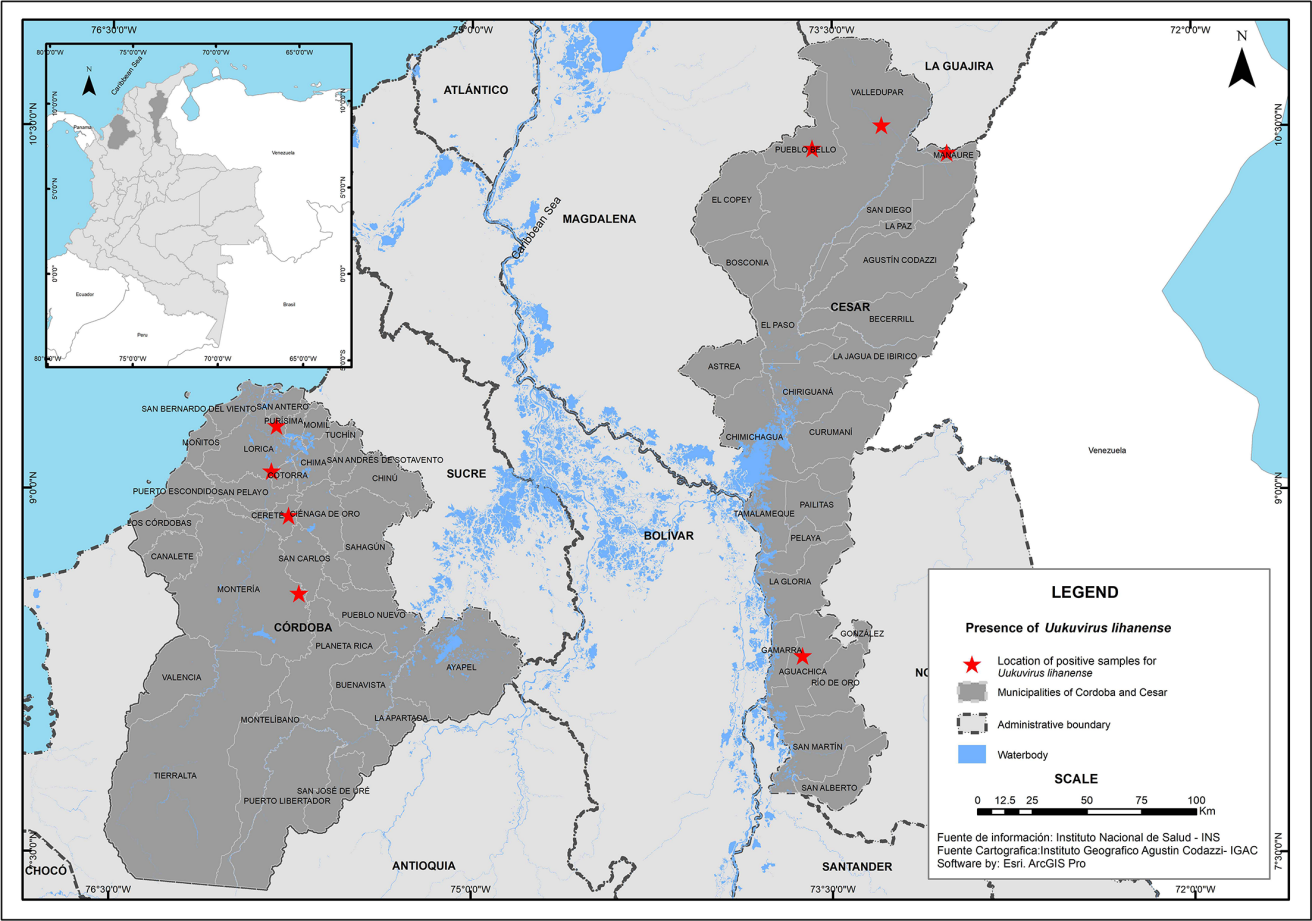
Geographic location of tick capture and municipalities sampled with positive results for LITV in the departments of Córdoba and Cesar.

Between 2022 and 2023, field trips were conducted in different locations in the departments of Córdoba and Cesar (Fig. 1). Seven hundred and sixty-six ticks were collected and processed. Ticks were collected directly from wild animals (snakes and iguanas) and domestic animals such as cattle, horses, sheep and dogs. Ticks were transported in liquid nitrogen to the laboratory and kept at −80 °C. Specimens were classified using taxonomic keys [22] and grouped by taxonomic genus, sex and stage, with a maximum of 12 individuals. Tick pools were macerated in 600 µl PBS, and 200 µl of the supernatant was transferred to a 22-µm filter. RNA was extracted from the filtrate using the GeneJet RNA Purification Kit from Thermo Scientific™. For sequencing, 13 tick pools were made, by species, genus, stage and geographic location.

### Library preparation and sequencing

The concentration and integrity of the RNA in the pools were measured using the kit IQ assay RNA and BR RNA with Qubit™ (Thermo Fisher Scientific™). Thirteen pools were processed using the MGIEasy RNA Library Prep PE-FLC 150 library preparation kit. The RNA was fragmented into products of ~250 nt. Then reverse transcription of the RNA, second-strand synthesis, end repair and adapter ligation were performed. PCR amplified the latter, and the library was quantified using fluorometry in Qubit™. Fragmentation was assessed using the Agilent Technologies™ Bioanalyzer. ssDNA circularization and DNA nanobead generation for high-throughput sequencing were performed using the MGI-G50 equipment, which generates a throughput of 560 million reads in 32 h (Shenzhen, China).

### Bioinformatics and phylogenetic analyses

Initially, the quality of the reads was verified using fastp [23]. The genome sequences of the different tick species analysed (*Rhipicephalus sanguineus* GenBank accession GCA_013339695.2, *Rhipicephalus microplus* GenBank accession GCF_013339725.1, *Dermacentor nitens* GenBank accession GCF_013339745.2 and *Amblyomma dissimile* GenBank accession GCA_023969395.1) were discarded through reference mapping using Bowtie2 v.2.5.0 [24]. *De novo* assembly was then performed to obtain contigs with MEGAHIT v.1.2.9 [25], and then the metatranscriptomes were submitted to accelerated blastx in diamond software using the ‘non-redundant’ database of National Center for Biotechnology Information (NCBI), the diamond output were meganized for taxonomic binning in MEGAN6 using a MEGAN mapping file (2022), following the recommendations of the diamond +megan for fast taxonomic analysis [26]. The metatranscriptome contigs matched with viruses were extracted and compared with the current ‘non-redundant’ database of proteins using the online tool blastx 2.14.1 [27]. The sequences identified as viral agents were annotated and translated using Prokka through the Galaxy online platform [28,29]. The deduced amino acid sequences of the viral transcripts identified in previous steps were aligned against the amino acid sequences of viral agents retrieved from GenBank [30] with the MAAFT server [31]. Maximum likelihood phylogenetic reconstructions were performed in IQ-TREE v2.2.2.6 with 1,000 ultrafast bootstrap, the best-fitting amino acid substitution model according to ModelFinder [32], phylogenetic trees were visualized in iTOL v.5 [33], and edited in Inkscape v.1.1 [34].

## Results

Only 9 of the 13 sequenced groups showed complete and partial fragments of the LITV genome. In the metatranscriptome of *R. microplus*, *D. nitens* and *A. dissimile*, complete and partial fragments of the L segment of LITV collected in the departments of Córdoba and Cesar were detected.

Only in the *R. microplus* species with code 12G from Cesar was the S segment of LITV found. No fragments of the M segment were detected. In the *R. sanguineus* species collected in Córdoba, LITV was not detected. Tick species with codes 11G, 29G and 117G were not included in the alignment because the amino acid sequences of the L segment were very short, with a size of 53 aa (Table 1).

**Table 1.** Pools of ticks collected for LITV by NGS

Code	Species	Sex/stage	Host	Location	Total reads	Total base (bp)	Contig		Segments of LITV	
							L	S	Segment l-RDRP	Segment S-nucleocapsid
7G	*A. dissimile*	♀	Iguana	Cesar	30.702.552	4.593.014	1.993		1.304 aa	
9G	*D. nitens*	♀	Bovine	Cesar	48.847.884	7.303.275	4.854		1.576 aa	
11G	*R. microplus*	♀	Bovine	Cesar	36.841.332	4.981.765	3.337		53 aa	
12G	*R. microplus*	♂	Bovine	Cesar	29.863.708	4.461.170	6.478	726	2.151 aa	146 aa
13G	*R. microplus*	N	Bovine	Cesar	33.981.760	5.056.211	6.486		2.151 aa	
26G	*R. microplus*	♀	Bovine	Córdoba	36.507.216	4.970.865	1.874		511 aa	
28G	*R. sanguineus*	♀	Dog	Córdoba	30.810.816	4.096.377				
29G	*R. microplus*	♂	Bovine	Córdoba	42.137.144	5.907.281	1813		53 aa	
117G	*R. microplus*	N	Bovine	Córdoba	27.668.692	3.708.193	873		53 aa	

aa, amino acid.

**Fig. 2.**
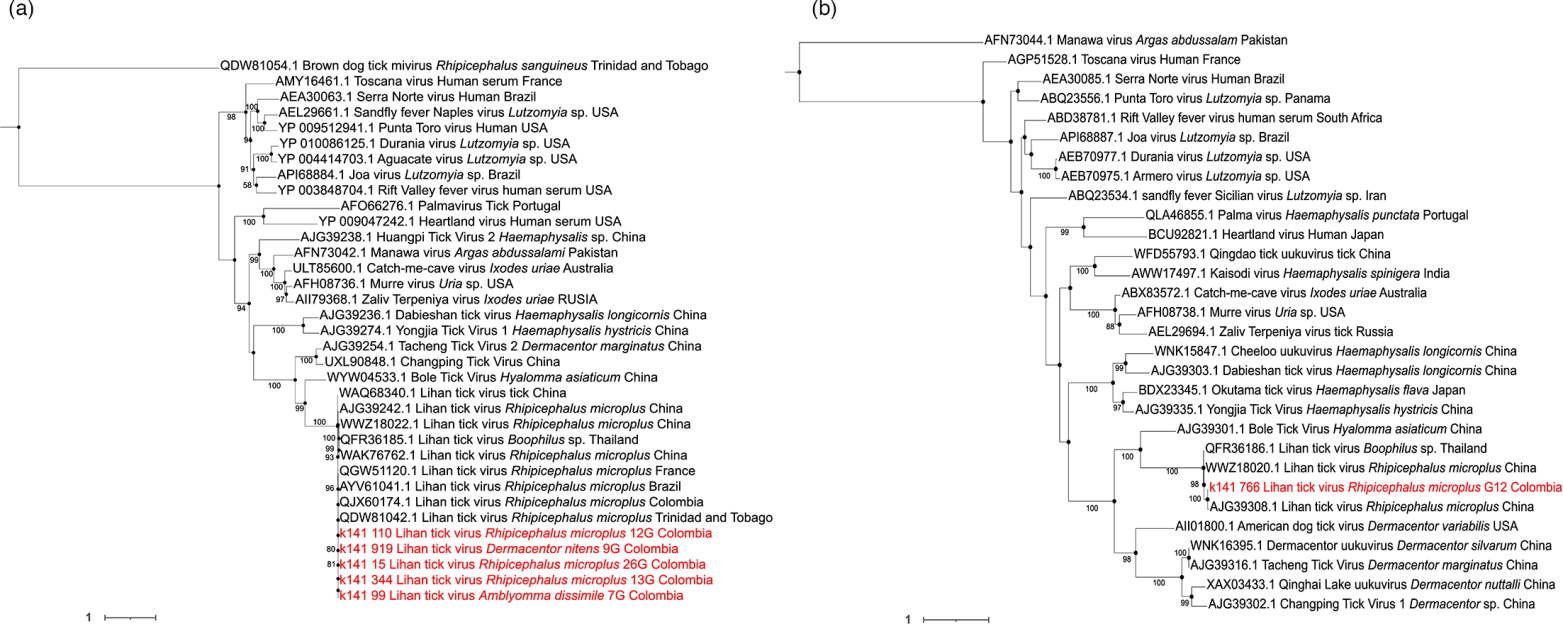
Phylogenetic constructs with amino acids from the L and S segments of LITV. (a) A Phylogenetic tree of the L segment encoding RdRp was constructed with 35 sequences (30 were downloaded from GenBank, and 5 were on their own). (b) A Phylogenetic tree of the S segment encoding the nucleocapsid has been built with 30 sequences (29 downloaded from GenBank and 1 own). These two trees were rooted with Brown dog tick mivirus (QDW81054.1) and Manawa virus (AFN73044). The sequences generated in this study are highlighted in red. The trees were constructed using the LG substitution model for the RdRp L and the nucleocapsid S segments.

Phylogenetic analysis of the L segment showed that LITV from this study is phylogenetically close to LITV (95% ultrafast bootstrap) from Trinidad and Tobago, Brazil, Colombia and the French Antilles. The aforementioned group of sequences LITV American sequences is related to LITV from the Hainan Province in southern China (Fig 2 and S1, available in the online Supplementary Material). In contrast, phylogenetic analysis of the S segment showed that the sequence reported in the study grouped with LITV detected in *R. microplus* from China and LITV in *Rhipicephalus* sp. from Thailand (Fig. 2).

Analysis of the two segments in blastp showed that the five amino acid sequences corresponding to the L segment have a percentage of identity between 99.86 and 99.32% with the LITV sequence reported in France, Trinidad and Tobago, and Brazil [1,35, 36]. Regarding the S segment, the amino acid sequence showed a percentage of identity of 99.32%, compared to the LITV sequence reported in Brazil [1].

## Discussion

LITV was identified from *R. microplus* and *D. nitens* collected from cattle and *A. dissimile* collected from iguanas in the Colombian Caribbean. The present work is the fifth report of LITV in Colombia and the first report of this virus in *A. dissimile*.

Currently, viruses of the *Uukuvirus* genus are not considered important for public health, although some studies have detected antibodies against some Uukuviruses in humans [10,37]. LITV belongs to the *Phenuiviridae* family, where the viral genus *Bandavirus* is found, which, in turn, has species of viruses that affect human health, such as *B. dabieense* and *B. heartlandense* transmitted by ticks [11,12]. It is known that the difference between *Bandavirus* and LITV is based on the fact that the M segment is absent [3,37]. In the present study, fragments of the M segment were not detected. These results are supported by several studies conducted in Colombia [5,16,18]. LITV was first identified in 2015 in *R. microplus* from China; the results demonstrated the diversity of viruses in ticks [3]. In 2018 in Brazil, almost complete sequences of LITV from the L and S segments were detected in *R. microplus*, and these results highlight that LITV has a high distribution frequency. In that study, LITV could not be cultured in vertebrate cell lines [1]. Later in 2019, in Trinidad and Tobago, the virome of *R. microplus*, *R. sanguineus* and *Amblyomma ovale* was analysed; only in *R. microplus* was LITV found [35].

In contrast, our study found LITV in *D. nitens* and *A. dissimile*, demonstrating the vast diversity of tick species that host LITV. In 2020, in the French Antilles, the two segments of LITV were detected in *R. microplus* from cattle. Phylogenetic analyses showed that the variant is related to the one found in China, suggesting a possible specificity with this tick species [36]. However, there are reports of LITV in *D. nitens* [17] and those found in our study in *A. dissimile*. In 2022 in China, LITV was detected in * R. microplus* and *R. sanguineus* collected from cattle and dogs; in that study, they suggested that LITV could present a low host restriction [38]. In the present study, it was also confirmed in *R. microplus*, *D. nitens* and *A. dissimile*; however, we could not detect LITV in *R. sanguineus*.

Regarding LITV studies in Colombia, the first report of LITV was in 2020 on *R. microplus* and *D. nitens*, collected in cattle and horses, respectively, in the department of Córdoba [17]. This finding differed from the first LITV detected in China in *R. microplus* [3]. The present study shows the wide range of tick species that carry this virus. In 2020, in the department of Antioquia, partial segments L and S were detected in *R. microplus* of cattle [16]. In 2021, LITV was detected in *R. microplus* of cattle and sheep in the middle Magdalena area. Orozco *et al*. [18] suggest this virus has a wide geographic distribution in *R. microplus* populations. However, in the present work, LITV was also found in *D. nitens* and *A. dissimile*. In addition, LITV was found in the Antioquia department of *Amblyomma patinoi* and *Amblyomma cajennense*. The results confirm this virus’s wide geographic distribution in several tick species [5]. It is essential to highlight that LITV has only been found in ticks, unlike other viruses, such as Jingmen, which are found in ticks [19] and mosquitoes [39].

Our results are important because LITV belongs to the *Uukuvirus* genus and is classified in the *Phenuiviridae* family [6,10]. Within this family, there are prominent representatives that affect human and animal health [11,12]. In addition, these findings reveal the circulation of LITV in a wide variety of tick species that can parasitize animals and humans.

In conclusion, this study identified LITV in the species *D. nitens* and *R. microplus* and is the first report of LITV in *A. dissimile*. Although LITV has not been considered important in public health, epidemiological surveillance studies in domestic and wild animals parasitized by the tick species involved are necessary. Additionally, cell culture and animal model studies must be implemented to determine the pathogenicity of LITV circulating in the region.

## Supplementary material

10.1099/acmi.0.000941.v3 Uncited Fig. S1.

## Abbreviations

LITV: *Uukuvirus lihanense*
NGS: next-generation sequencing
