# Supplementary material for: Detection and genetic characterization of Uukuvirus lihanense (Uukuvirus, Phenuiviridae) in hard ticks from the Colombian Caribbean
Source: Access Microbiol. 2025 Oct 13;7(10):000941.v3. doi: 10.1099/acmi.0.000941.v3 (PMC12517356; doi:10.1099/acmi.0.000941.v3)
Supplement: Uncited Fig. S1. [file acmi-7-00941-s001.pdf]

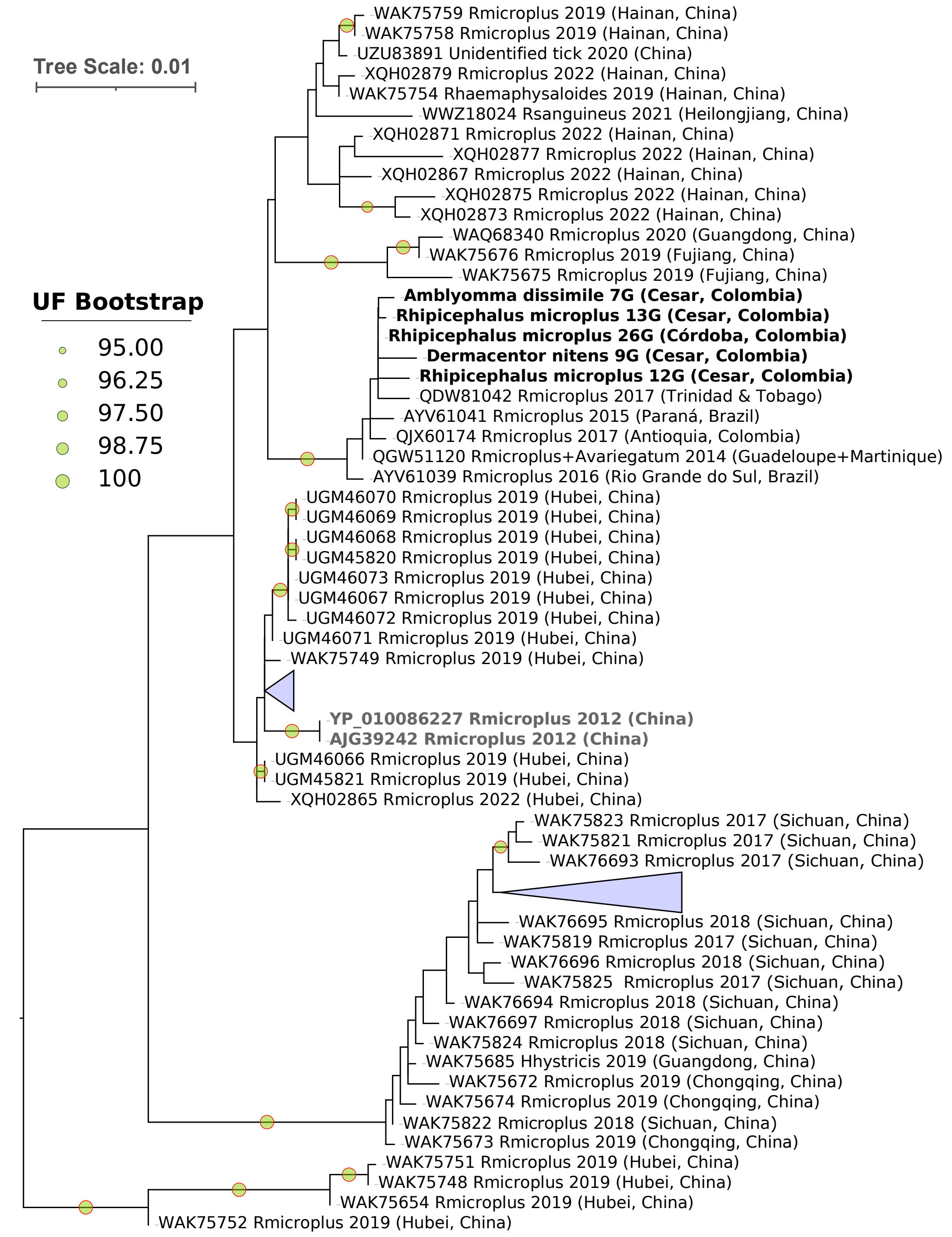

Supplementary ML phylogenetic tree. The ML tree was calculated in IQ-Tree using the aminoacidic sequences of Lihan Tick viruses (Uukuvirus lihanense) reported in INCBI, clades below 25% of ultrafast bootstrap were collapsed.
